# Rapid Increase in Pertactin-deficient *Bordetella pertussis* Isolates, Australia

**DOI:** 10.3201/eid2004.131478

**Published:** 2014-04

**Authors:** Connie Lam, Sophie Octavia, Lawrence Ricafort, Vitali Sintchenko, Gwendolyn L. Gilbert, Nicholas Wood, Peter McIntyre, Helen Marshall, Nicole Guiso, Anthony D. Keil, Andrew Lawrence, Jenny Robson, Geoff Hogg, Ruiting Lan

**Affiliations:** University of New South Wales, Sydney, New South Wales, Australia (C. Lam. S. Octavia, L. Ricafort, R. Lan);; University of Sydney, Sydney (V. Sintchenko, G.L. Gilbert);; Westmead Hospital, Sydney, (V. Sintchenko, N. Wood, P. McIntyre);; University of Adelaide, Adelaide, South Australia, Australia (H. Marshall);; Institut Pasteur, Paris, France (N. Guiso);; Princess Margaret Hospital for Children, Perth, Western Australia, Australia (A.D. Keil);; Women’s and Children’s Hospital, Adelaide (A. Lawrence);; Sullivan Nicolaides Pathology, Brisbane, Queensland, Australia (J. Robson);; University of Melbourne, Parkville, Victoria, Australia (G. Hogg)

**Keywords:** Bordetella pertussis, whooping cough, bacteria, outbreaks, pertactin, evolution, immunization, vaccination, vaccine, Australia

## Abstract

Acellular vaccines against *Bordetella pertussis* were introduced in Australia in 1997. By 2000, these vaccines had replaced whole-cell vaccines. During 2008–2012, a large outbreak of pertussis occurred. During this period, 30% (96/320) of *B. pertussis* isolates did not express the vaccine antigen pertactin (prn). Multiple mechanisms of prn inactivation were documented, including IS*481* and IS*1002* disruptions, a variation within a homopolymeric tract, and deletion of the *prn* gene. The mechanism of lack of expression of prn in 16 (17%) isolates could not be determined at the sequence level. These findings suggest that *B. pertussis* not expressing prn arose independently multiple times since 2008, rather than by expansion of a single prn-negative clone. All but 1 isolate had *ptxA1*, *prn2*, and *ptxP3*, the alleles representative of currently circulating strains in Australia. This pattern is consistent with continuing evolution of *B. pertussis* in response to vaccine selection pressure.

*Bordetella pertussis* is the gram-negative coccobacillus that causes the respiratory disease pertussis, also known as whooping cough. The incidence of pertussis infection and related deaths decreased dramatically after implementation of immunization with a whole-cell vaccine (WCV) during the 1950s. Because of side effects of WCV, such as high rates of fever and local reactions, and variable efficacy of WCVs, a less reactogenic acellular vaccine (ACV) was developed in the 1980s. ACVs have now replaced WCVs in many industrialized countries for primary and booster vaccinations against pertussis.

Although ACV formulations differ in the number of component pertussis antigens, the vaccine used in Australia contains pertussis toxin (ptx), pertactin (prn), and filamentous hemagglutinin (fha). A 5-component (ptx, prn, fha, fimbrial antigen [fim]2, and fim3) ACV is used for short periods in some regions (*1*). ACVs were introduced for the fourth and fifth doses in most states in Australia during 1997 and for all doses during 1999 (Figure 1). South Australia introduced ACVs for all doses in 1997. The current vaccination schedule for pertussis comprise 3 primary doses of ACV at 2, 4, and 6 months of age, and a booster vaccination at 4 years of age. A booster vaccination with ACV at 18 months of age, which was introduced in 1985, was removed from the National Immunization Program in Australia in 2003, and an adult-formulated ACV was introduced for children at 12–17 years of age in school-based programs in 2004 (*2*,*3*).

**Figure 1:**
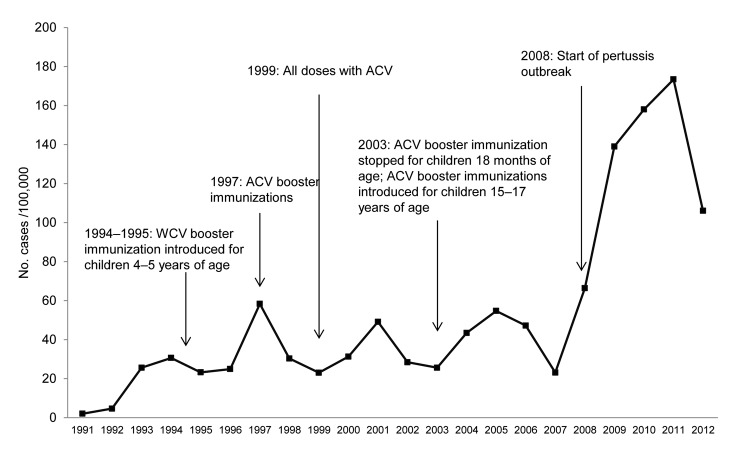
Pertussis cases/100,000 population in Australia, 2008–20012, since mandatory reporting was instituted in 1991 and changes to pertussis vaccination schedule, including introduction of whole-cell vaccine (WCV) booster vaccinations for 4–5-year-old children in 1994–1995 and introduction of acellular vaccine (ACV) booster vaccinations in 1997. By 1999–2000, ACVs were used for all pertussis vaccinations. In 2003, the booster vaccinations for children 18 months of age was removed and replaced with a booster vaccination for children 15–17 years of age (*3*).

Since 1991, data on reported pertussis cases show that outbreaks occurred in Australia in 1996–1997, 2001, and 2004, and a series of outbreaks occurred in different regions starting in 2008 (Figure 1) (*2*,*3*). Multiple factors probably contributed to the resurgence of pertussis in high-income countries that had long-standing pertussis immunization programs. These factors include waning immunity (exacerbated by the change from WCVs to ACVs and, in Australia, cessation of the booster vaccination at 18 months of age) and increased use of more sensitive diagnostic tests, such PCR (*4*).

An additional possible contributing factor is evolution of *B. pertussis* through vaccine-driven adaptation (*5*). The most prominent recent changes in circulating *B. pertussis* strains are polymorphisms within genes encoding 2 of the 3 main virulence factors (ptx and prn) contained in the vaccine. Variations have also been reported in *ptxP*, the promoter of the *ptx* operon (*6*). In Australia, we have shown by single nucleotide polymorphism (SNP) typing that among *B. pertussis* isolates, *ptxP3*–containing strains predominate (*7*), and these strains belong to SNP cluster I (*8*,*9*).

Surveillance of recent *B. pertussis* isolates in several countries has identified *prn* deletions and gene disruptions, which lead to lack of expression of mature prn (*10*–*13*). This protein is a 69-kDa adhesin that aids *B. pertussis* attachment to epithelial cells and is one of the most polymorphic virulence genes within *B. pertussis* (it has 13 documented alleles) (*5*). SNPs and differences in the number of amino acid (GGFGP and PQP) repeats contribute to variation within the *prn* gene; variations are usually limited to 2 regions known as region 1 and region 2.

In this study, we identified *B. pertussis* isolates that do not express prn (prn negative) from a set of isolates collected in Australia during 1997–2012. We also characterized the causes of their lack of expression and evaluated trends in the proportion of prn-negative isolates over this period.

## Methods

### Bacterial Strains and Growth

A total of 453 *B. pertussis* isolates were available for this study; 133 isolates collected during 1997–2008 and 194 collected during 2008–2010 have been described (*9*). A total of 126 additional isolates collected from Westmead Hospital (Sydney, New South Wales, Australia) and Princess Margaret Hospital for Children (Perth, Western Australia, Australia) during 2011–2012 were also included this study. Although specific clinical information about the source of isolates was not available, isolates were obtained from patients who lived in large urban areas and who had PCR-confirmed pertussis infections. The number of available isolates in 2011–2012 was relatively small because several participating laboratories discontinued pertussis culture in favor of only direct PCR testing.

All *B. pertussis* isolates were grown on Bordet Gengou agar (Becton Dickinson, Sparks, MD, USA) supplemented with 10% defibrinated horse blood (Oxoid, Basingstoke, UK) at 37°C for 3–5 days before subculture and incubation at 37°C for 24 h. All cultures were examined for hemolytic activity indicating expression of the virulent (Bvg+) phase before being collected and resuspended in saline to an optical density at 650 nm = 1 for Western immunoblotting.

### Western Immunoblotting

The ptx, prn, and fha proteins were detected by Western immunoblotting as described (*10*,*11*,*14*). Bacterial suspensions were mixed with Laemmli buffer containing 5% β-mercaptoethanol and boiled for 5 min. Proteins separated by sodium dodecyl sulfate–polyacrylamide gel electrophoresis were transferred to a polyvinylidene difluoride membrane at 100 V for 1 hr. Membranes were blocked with 5% (wt/vol) skim milk powder in wash buffer for 1 hr and incubated overnight with mouse polyclonal antibodies against ptx, fha, and prn diluted 1:1,000 with Tris-buffered saline (TBS) containing 1% Tween 20. After 3 washes with TBS containing 1% Tween 20, membranes were incubated for 1 h with sheep antimouse monoclonal antibodies in TBS plus 5% skim milk and 0.1% Tween 20. Antigen–antibody complexes were visualized by chemiluminescence on a LAS3000 imager (Fujifilm, Tokyo, Japan). The minimum detectable amount with this method was 1 ng of specific protein.

### Genotyping and *prn* Gene Sequencing

Isolates were genotyped for *fim3*, *prn*, and *ptxP* alleles as described (*7*–*9*). Isolates that had not already been typed were characterized by SNP cluster and SNP profile as described by Octavia et al. (*8*), multilocus variable number tandem repeat analysis (MLVA) as described by Kurniawan et al. (*1*) typing of *prn, fim3*, and *ptxP* alleles (*6*,*15*). Relationships among SNP profiles and clusters were defined by Octavia et al. (*8*) and are shown in Technical Appendix Figure 1.

For isolates that did not express prn, overlapping primers reported by Fry et al. (*16*) were used to amplify a predicted 2,869-bp region that included the signal peptide region and the *prn* gene. The *prn* promoter region was also sequenced to detect any changes. Each PCR mixture contained ≈30 ng DNA, 10 mmol/L Tris-HCl (pH 8.3), 50 mmol/L KCl, 2.5 mmol/L MgCl_2_, 100 µmol/L of each deoxynucleotide, 10 pmol/L of each primer, 2.5 units of *Taq* polymerase, and milliQ water (Millipore, Billerica, MA, USA). Products were then sequenced on an Automated DNA Sequence Analyzer ABI3730 (Applied Biosystems, Foster City, CA, USA) to determine the complete *prn* gene, which included region 1 and region 2. All sequences were aligned against *prn* gene sequences identified by Mooi et al. (*17*). 

## Results

### Identification and Distribution of *B. pertussis* Not Expressing prn

The 320 *B. pertussis* isolates obtained during 2008–2012 were from 5 states in Australia: New South Wales (116 isolates), Queensland (37), South Australia (47), Victoria (30), and Western Australia (90). All 96 (30) isolates identified by Western immunoblot as not expressing prn were obtained after 2008. Examples of Western immunoblots are shown in online Technical Appendix Figure 2. The other 133 isolates obtained before 2008 expressed prn and were from SNP clusters I and II or were unclustered. The distribution of prn-negative isolates in individual states is shown in the Table. Only isolates from Western Australia and New South Wales were available for all years during 2008–2012; no isolates were available from South Australia or Victoria during 2011–2012 or from Queensland during 2008–2009 and 2012. All isolates expressed ptx and fha.

**Table:** Distribution of pertactin-positive and protactin-negative *Bordetella pertussis* isolates in 5 states, Australia, 2008–2012*

Year	State														
	New South Wales			Queensland			South Australia			Victoria			Western Australia		
	No. pos	No. neg	% Neg	No. pos	No. neg	% Neg	No. pos	No. neg	% Neg	No. pos	No. neg	% Neg	No. pos	No. neg	% Neg
2008	18	0	0	–	–	–	13	2	13	1	0	0	5	0	0
2009	52	0	0	–	–	–	17	9	35	10	1	9	18	2	10
2010	8	6	43	3	6	67	5	1	17	17	1	6	14	1	7
2011	6	17	74	21	7	25	–	–	–	–	–	–	8	15	65
2012	2	7	78	–	–	–	–	–	–	–	–	–	6	21	78

*Pos, positive; Neg, negative; –, no isolates were obtained.

**Figure 2:**
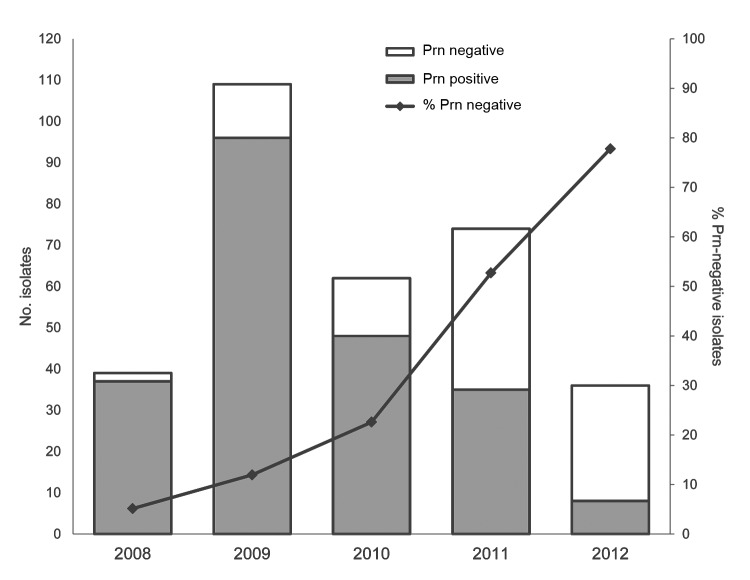
Number and percentage of pertactin (Prn)–negative *Bordetella pertussis* isolates in Australia, 2008–2012. During this period, 320 *B. pertussis* isolates obtained in New South Wales, Queensland, South Australia, Victoria, and Western Australia were identified as expressing prn or not expressing prn by using Western immunoblotting. The increasing percentage of prn-negative isolates each year during 2008–2012 was 5%, 12%, 23%, 53%, and 78% respectively. Data for individual states and years can be found in the Table. Gray bars indicate number of isolates expressing prn, and white bars indicate number of isolates not expressing prn. Error bars indicate 95% CIs.

The prn-negative strains were first identified in isolates collected in 2008, when they made up 5% (2/39) of the isolates. By 2012, the proportion of prn-negative isolates had increased to 78% (28/36) (Figure 2). In Western Australia and New South Wales, where isolates were available for all years, there was a progressive increase from 3% in 2009 to 78% in 2012 (Technical Appendix Figure 3). Lack of isolates from Queensland, Victoria, and South Australia in various years was related to changes in laboratory practice (cultures no longer obtained) or decreased numbers in a post-epidemic period, rather than any systematic differences in collection. It is unlikely that different patterns of circulating *B. pertussis* differed in these regions.

The increase in prn-negative isolates during 2011–2012, in comparison with 2008, was significant (p<0.05, by Fisher exact test with multiple test correction). All but 1 prn-negative isolate had the *ptxA1*, *prn2*, and *ptxP3* alleles and belonged to SNP cluster I; the exception, L1378, had *ptxA1* and *prn1* but not *ptxP3*, and was not assigned to any SNP cluster. In addition, the prn-negative isolates had new MLVA types that were closely related to MT27 and MT114, both of which are currently circulating in Australia (*9*), although MT27 still predominates.

### Sequence Analysis of *prn* Gene of prn-deficient Isolates

**Figure 3:**
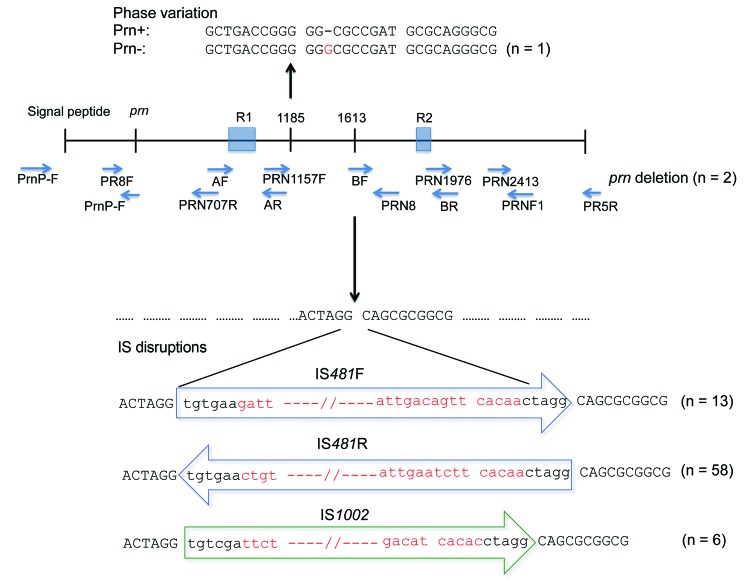
Variations in protactin (*prn*) gene of prn-negative *Bordetella pertussis* isolates, Australia, 2008–2012, Ninety-six *B. pertussis* isolates were identified as prn negative. Eighty of these isolates had 1 of 4 mechanisms of *prn* disruption: IS*481* (in forward and reverse directions) and IS*1002,* which were inserted at the ACTAGG motif within *prn*, or an extended homopolymeric tract of G residues (n = 1). Lower case letters indicate residues that are conserved in all IS disruptions, and red letters indicate differences in IS disruptions. Positions of nucleotides have been numbered relative to the first start codon of sequence AJ011092 (*17*). The *prn* gene of 2 isolates was not amplified by PCR with a combination of primers from published studies (*15**–**19*), which indicated a deletion of the entire gene. Sixteen isolates that had no gene disruptions were also observed.

Mechanisms of disruption, identified by sequencing the *prn* region, including the signal peptide, of 80/96 prn-negative isolates, are shown in Figure 3. Seventy-seven (82%) isolates had IS elements located between region 1 and region 2; in *prn*, a 1049-bp IS*481* was inserted in the forward direction in 13 isolates and in the reverse direction in 58 isolates. A 1,037-bp IS*1002* was inserted in the forward direction in 6 isolates, which has not been described in the *prn* region. All IS element disruptions were at position 1613 and were flanked by a 6-bp repeat (ACTAGG) at the 5′ end and AGGCAG at the 3′ end (Figure 3).

One isolate had no IS within *prn* but had an additional guanine residue at position 1185 between region 1 and region 2, which resulted in a stop codon at amino acid position 749. Two isolates from South Australia that had SNP profile SP13 were nontypeable. For *prn*; multiple pairs of PCR primers specific for the *prn* gene (*15*–*19*) failed to amplify a product, which indicated deletion of the entire gene. IS disruptions, deletions, or other variations were not detected in *prn* or the *prn* promoter region of 16 prn-negative isolates. Details of the 96 prn-negative isolates, including individual *prn*, *fim*, and *ptxP* alleles, SP, MLVA type, and mechanism of prn disruption, are shown in the online Technical Appendix Table.

## Discussion

In the 2 regions of Australia where isolates were available for all years during 2008–2012, prn-negative *B. pertussis* isolates increased from >10% to ≈80% of *B. pertussis* isolates over this period. Prn-negative strains have been isolated in several countries that have high coverage for vaccination but have not been shown to constitute such a high proportion of circulating *B. pertussis* (*12*,*13*,*20*,*21*). Japan was the first country to implement ACVs against pertussis in 1981, and the proportion of prn-negative isolates reported from countrywide surveillance during 2005–2009 was 32% (18/57). In France, where ACVs have been used since 1998, originally as booster vaccinations, and then for all doses since 2002 (*11*), ptx-negative and fha-negative isolates were first obtained in 2003, although only prn-negative isolates have increased and were reported to make up 13.3% of 120 isolates analyzed in 2011 (*10*).

The prn-negative *B. pertussis* isolates have also been identified in Finland and the United States (*13*,*21**,**22*). The United States introduced ACVs as booster vaccinations in 1991, but not until 1997 were all 5 primary doses replaced with ACVs (*23*). Although Finland replaced WCVs with ACVs at a later time (booster vaccinations in 2003 and primary vaccinations in 2005), both countries detected prn-negative isolates during 2011–2012. Long-term temporal analysis has not been performed to determine whether such isolates are increasing over time.

In comparison, until 2001 and 2009, respectively, Russia (*24*) and Senegal (*25*), which currently use only WCVs, have not reported prn-negative isolates. However, it is difficult to draw a definitive conclusion on the correlation of timing of emergence of prn-negative strains with timing of introduction of ACVs. Extensive analysis of isolates from earlier years from different countries would be required.

Multiple mechanisms of lack of expression of prn have been reported (*11*,*12*). Insertion of IS*481* into the *prn* gene in either the forward or reverse direction was still the main mechanism of disruption (73.9%). This disruption occurred at the same conserved site identified in 3 isolates from the United States (nt position 1613) (*21*) and 9 isolates from Japan (nt position 1598) (*12*). The 15-bp difference in position is caused by an additional GGFGP repeat in *prn2* in the isolates in our study and those from the United States, compared with those from Japan, which have *prn1.*

Six isolates in our study had an additional IS*1002* disruption at nucleotide position 1613 (Figure 2), which confirmed that the 6-bp repeat site flanking IS elements is conserved (*26*). The lower number of isolates with IS*1002* disruptions could be caused by fewer copies of IS*1002* than IS*481* in the genome (6 for IS*1002* in Tohama I compared with 238 for *IS481*). Disruption of virulence genes by IS*1002* has been reported; it can result in *B. pertussis* isolates not expressing O antigen (*27*). All isolates in this study that had the IS*1002* disruption were collected in 1 state in Australia (New South Wales) and might have arisen from a single outbreak. More isolates are needed to determine whether this finding is indicative of an expanding clone.

Another mechanism of disruption is an 84-bp deletion of the signal peptide (nucleotide position 26). This deletion was observed in 24 isolates from Japan (*12*) and 2 isolates from Finland (*13*) but was not observed in any of the isolates in our study. However, 2 of the isolates apparently had the entire *prn* gene deleted.

In 1 isolate (L1502), an additional G residue in a homopolymeric tract of G residues resulted in a downstream stop codon. Truncations caused by stop codons in the *prn* gene were reported in 7 isolates from the United States (*21*), but they were at nucleotide position 1273 and the actual base change was not specified. Phase variation has been associated with variation in other *B. pertussis* genes, including *fim2*, *fim3*, *fimX*, and *bapC* (*28*), and is a common mechanism of phase variation in other pathogenic microorganisms (*29**,**30*).

A large proportion (17%, 16/96) of prn-negative isolates had no sequence change detected in the *prn* gene or its promoter upstream, which indicated that other mechanisms must have been responsible for inactivating prn expression. These 16 isolates belong to 3 SNP profiles; 8, 7, and 1 isolates belonging to SP13, SP14, and SP18, respectively, which suggests 3 independent inactivating events. Inactivation of expression could have occurred at the transcriptional or translational level. Our preliminary investigations showed that 3 of these prn-negative isolates produced *prn* gene transcripts. A consequence of prn inactivation without sequence variation of the *prn* gene is that it can be detected only at the protein level. Until mechanisms are identified, culturing of isolates will still be needed to monitor *B. pertussis*.

The increase in isolates that do not express a specific antigen has been documented only recently in Australia and other countries that use ACVs. The predominant isolates we identified are from SP13, SP14, or SP16, and all but 1 had the *ptxA1*, *prn2*, *ptxP3* genotype. We have also shown that isolates with different SNP profiles can be affected by the same IS disruptions, and conversely, different IS disruptions can affect isolates with the same SNP profiles.

Most of the recently isolated prn-negative strains from the United States have the *prn2* allele, which has been the predominant type since the 1990s (*21**,**22*). However, mutations causing inactivation of expression of the *prn* gene differ from those reported in this study and elsewhere. Prn-negative isolates characterized by Otsuka et al. (*12*) had the *prn1*, *ptxA2*, *ptxP1* genotype and were from MT186 or related MT194 or MT226. Our previous analysis showed that MT186 belongs to SNP cluster V; this type is unrelated to isolates examined in the current study, which belong to SNP cluster I (Technical Appendix Figure 1), but was affected by the same IS disruption mechanisms. Two isolates from Finland that had *prn1* were also reported to be prn negative because of deletions, although *prn2* is the current predominant allele (*13*). Thus, the combination of SNP typing, antigen gene typing, and *prn* gene disruption mechanisms clearly demonstrates that isolates that do not express the *prn* gene from Australia and other countries do not belong to the same clone and that the recent almost simultaneous appearance and expansion of prn-negative isolates in several countries were independent events rather than global spread of a single clone.

The multiple origins of prn-negative isolates also point strongly to selective pressure on the bacterium. Therefore, it is conceivable that these prn-negative isolates are more likely to evade a vaccine-induced immune response. However, the relative contribution of prn to pertussis disease has not been clearly established. Various studies using *prn* mutants have shown that mutants that do not express prn do not colonize mouse lungs as well as isolates that express prn (*31*) but were more invasive in epithelial cells and persist for a longer period (*32*). The prn-negative strains have a greater growth advantage in vitro than their Prn-positive counterparts (*12*). This growth advantage can be beneficial in maintaining a high level of transmissibility between hosts, which is consistent with increasing numbers of infections with prn-negative isolates identified in Australia and elsewhere.

Whether these isolates have greater or lesser virulence than prn-positive strains is unclear. In contrast to lack of production of ptx, loss of prn does not seem to affect *B. pertussis* lethality in mice, possibly because of the range of autotransporters within *B. pertussis* that can compensate for the role of prn (*10*). In a retrospective study, no differences were found in severity of symptoms or duration of hospitalization between infants infected with prn-positive and prn-negative strains in France (*20*); the only major difference observed was the longer period from onset of pertussis symptoms to time of hospitalization among infants whose *B. pertussis* isolate was prn negative. Regardless of prn expression, vaccination reduced the severity of disease and the likelihood of being admitted to intensive care, which suggests that even an incomplete course of primary vaccination provides some protection against severe pertussis (*20*).

The results in this study highlight the emerging trend of prn-deficient *B. pertussis* isolates circulating in Australia. In addition to changes observed in *prn*, *ptxA*, *ptxP*, and *fim* genes of currently circulating strains, this study and other studies have reported the increasing prevalence of isolates not expressing prn in many countries that have a high uptake of ACV. The overall effect of lack of expression of an antigen on herd immunity is unknown. Emergence of prn-negative isolates is a relatively recent phenomenon that has affected currently circulating *B. pertussis* isolates. Whether strains not expressing prn continue to increase locally or globally and affect vaccine effectiveness and bacterial pathogenicity is unknown. Continued monitoring of genotypic and phenotypic properties of *B. pertussis* is required to better understand the effects of vaccination on the evolution of the organism.

Technical Appendix Characteristics of Pertactin-deficient *Bordetella pertussis* Isolates, Australia, 2008–2012.
