# Supplementary material for: Rapid Increase in Pertactin-deficient Bordetella pertussis Isolates, Australia
Source: Emerg Infect Dis. 2014 Apr;20(4):626–33. doi: 10.3201/eid2004.131478 (PMC3966384; doi:10.3201/eid2004.131478)
Supplement: Technical Appendix — Characteristics of Pertactin-deficient Bordetella pertussis Isolates, Australia, 2008–2012. [file 13-1478-Techapp-s1.pdf]

Article DOI: http://dx.doi.org/10.3201/eid2004.131478

Rapid Increase in Pertactin-deficient *Bordetella pertussis* Isolates, Australia

Technical Appendix

Characteristics of Pertactin-deficient *Bordetella pertussis* Isolates, Australia, 2008–2012

Technical Appendix Table. Characteristics of perlactin-negative isolates of *Bordetella pertussis* from 5 states of Australia, 2008–2012*

Strain	Year	SP	MLVA†	Disruption‡	*prn*	*fim*	*Ptx*
South Australia							
L1277	2008	14	New 2 (27)	No ISE	*prn2*	A	*ptxP3*
L1283	2009	14	27	IS*481*R	*prn2*	A	*ptxP3*
L1287	2009	14	New 2 (27)	No ISE	*prn2*	A	*ptxP3*
L1290	2009	14	27	IS*481*R	*prn2*	A	*ptxP3*
L1294	2009	14	27	IS*481*R	*prn2*	A	*ptxP3*
L1295	2009	13	27	No ISE	*prn2*	B	*ptxP3*
L1297	2009	14	27	IS*481*R	*prn2*	A	*ptxP3*
L1308	2009	13	28	No ISE	*prn2*	B	*ptxP3*
L1314	2009	13	New 14 (214)	No ISE	*prn2*	A	*ptxP3*
L1326	2008	13	New 2 (27)	No ISE	*prn2*	A	*ptxP3*
Victoria							
L1333	2009	14	New 7 (72)	IS*481*F	*prn2*	B	*ptxP3*
L1352	2010	14	18	No ISE	*prn2*	B	*ptxP3*
Western Australia							
L1361	2009	13	New 3 (214)	IS*481*R	*prn2*	A	*ptxP3*
L1371	2009	13	New 3 (214)	IS*481*R	*prn2*	A	*ptxP3*
L1378	2010	18	New 8	No ISE	*prn1*	A	Not *ptxP3*
L1749	2011	13	27	IS*481*R	*prn2*	A	*ptxP3*
L1751	2011	13	27	IS*481*R	*prn2*	A	*ptxP3*
L1752	2011	13	27	IS*481*F	*prn2*	A	*ptxP3*
L1753	2011	13	27	IS*481*F	*prn2*	A	*ptxP3*
L1756	2011	13	27	IS*481*R	*prn2*	A	*ptxP3*
L1757	2011	14	18	No ISE	*prn2*	B	*ptxP3*
L1759	2011	13	27	IS*481*R	*prn2*	A	*ptxP3*
L1762	2011	14	18	No ISE	*prn2*	B	*ptxP3*
L1763	2011	13	27	IS*481*R	*prn2*	A	*ptxP3*
L1764	2011	13	27	IS*481*F	*prn2*	A	*ptxP3*
L1766	2011	13	27	IS*481*R	*prn2*	A	*ptxP3*
L1767	2011	13	27	IS*481*R	*prn2*	A	*ptxP3*
L1768	2011	13	27	IS*481*R	*prn2*	A	*ptxP3*
L1769	2011	13	27	IS*481*R	*prn2*	A	*ptxP3*
L1771	2011	14	18	No ISE	*prn2*	B	*ptxP3*
L1772	2012	13	27	IS*481*R	*prn2*	A	*ptxP3*
L1773	2012	13	27	IS*481*R	*prn2*	A	*ptxP3*
L1775	2012	13	27	IS*481*R	*prn2*	A	*ptxP3*
L1776	2012	13	27	IS*481*R	*prn2*	A	*ptxP3*
L1777	2012	13	27	IS*481*R	*prn2*	A	*ptxP3*
L1778	2012	13	27	IS*481*R	*prn2*	A	*ptxP3*
L1779	2012	13	27	IS*481*R	*prn2*	A	*ptxP3*
L1782	2012	13	27	IS*481*R	*prn2*	A	*ptxP3*
L1783	2012	13	27	IS*481*R	*prn2*	A	*ptxP3*
L1784	2012	13	27	IS*481*R	*prn2*	A	*ptxP3*
L1786	2012	13	27	IS*481*R	*prn2*	A	*ptxP3*
L1787	2012	13	27	IS*481*R	*prn2*	A	*ptxP3*
L1788	2012	13	27	IS*481*R	*prn2*	A	*ptxP3*
L1789	2012	13	27	IS*481*F	*prn2*	A	*ptxP3*
L1790	2012	13	27	IS*481*R	*prn2*	A	*ptxP3*
L1791	2012	13	27	IS*481*R	*prn2*	A	*ptxP3*
L1793	2012	13	27	IS*481*F	*prn2*	A	*ptxP3*
L1795	2012	13	New 3 (214)	IS*481*R	*prn2*	A	*ptxP3*
L1796	2012	13	27	IS*481*R	*prn2*	A	*ptxP3*
L1797	2012	13	27	IS*481*F	*prn2*	A	*ptxP3*
L1798	2012	13	27	IS*481*R	*prn2*	A	*ptxP3*
New South Wales							

Strain	Year	SP	MLVA†	Disruption‡	*prn*	*fim*	*Ptx*
L1421	2010	13	New 19 (114)	IS*1002*	*prn2*	A	*ptxP3*
L1422	2010	13	New 19 (114)	IS*1002*	*prn2*	A	*ptxP3*
L1424	2010	16	New 19 (114)	IS*481*R	*prn2*	B	*ptxP3*
L1425	2010	16	New 19 (114)	IS*481*R	*prn2*	B	*ptxP3*
L1429	2010	16	New 19 (114)	IS*481*R	*prn2*	B	*ptxP3*
L1432	2010	16	New 22 (114)	IS*481*R	*prn2*	B	*ptxP3*
L1492	2011	16	27	IS*481*R	*prn2*	B	*ptxP3*
L1493	2011	13	27	IS*481*R	*prn2*	A	*ptxP3*
L1495	2011	16	27	IS*481*F	*prn2*	B	*ptxP3*
L1496	2011	13	27	IS*1002*	*prn2*	A	*ptxP3*
L1498	2011	16	27	IS*481*R	*prn2*	B	*ptxP3*
L1499	2011	13	27	IS*481*F	*prn2*	A	*ptxP3*
L1501	2011	13	NA	IS*1002*	*prn2*	A	*ptxP3*
L1502	2011	13	27	HPT-G	*prn2*	A	*ptxP3*
L1503	2011	13	27	IS*481*R	*prn2*	A	*ptxP3*
L1504	2011	13	27	IS*1002*	*prn2*	A	*ptxP3*
L1505	2011	16	New 23	IS*481*R	*prn2*	A	*ptxP3*
L1506	2011	16	27	IS*481*R	*prn2*	B	*ptxP3*
L1507	2011	13	27	IS*481*R	*prn2*	A	*ptxP3*
L1508	2011	16	27	IS*481*R	*prn2*	B	*ptxP3*
L1529	2011	13	27	IS*481*R	*prn2*	B	*ptxP3*
L1532	2011	13	New 2 (27)	No ISE	*prn2*	A	*ptxP3*
L1554	2011	13	27	IS*481*R	*prn2*	A	*ptxP3*
L1658	2012	13	27	IS*1002*	*prn2*	A	*ptxP3*
L1659	2012	14	18	No ISE	*prn2*	B	*ptxP3*
L1661	2012	13	27	IS*481*R	*prn2*	A	*ptxP3*
L1662	2012	13	27	IS*481*R	*prn2*	A	*ptxP3*
L1663	2012	13	27	IS*481*F	*prn2*	A	*ptxP3*
L1664	2012	16	27	IS*481*R	*prn2*	B	*ptxP3*
L1665	2012	16	27	IS*481*R	*prn2*	B	*ptxP3*
Queensland							
L1512	2010	13	27	IS*481*F	*prn2*	A	*ptxP3*
L1513	2010	13	22	No ISE	*prn2*	A	*ptxP3*
L1514	2010	13	27	IS*481*R	*prn2*	A	*ptxP3*
L1515	2010	13	27	IS*481*R	*prn2*	A	*ptxP3*
L1516	2010	13	27	IS*481*R	*prn2*	A	*ptxP3*
L1519	2010	13	27	IS*481*F	*prn2*	A	*ptxP3*
L1526	2011	13	27	IS*481*F	*prn2*	A	*ptxP3*
L1534	2011	13	27	IS*481*R	*prn2*	A	*ptxP3*
L1536	2011	13	New 2 (27)	No ISE	*prn2*	A	*ptxP3*
L1539	2011	15	27	IS*481*R	*prn2*	B	*ptxP3*
L1540	2011	13	27	IS*481*R	*prn2*	A	*ptxP3*
L1553	2011	13	27	IS*481*R	*prn2*	A	*ptxP3*
L1558	2011	13	27	No ISE	*prn2*	A	*ptxP3*
Untypeable prn, South Australia							
L1301	2009	13	New 2 (27)	NA	Untypeable	A	*ptxP3*
L1304	2010	13	27	NA	Untypeable	A	*ptxP3*

*SP,single-nucleotide polymorphism profile; MLVA, multilocus variable number tandem repeat analysis; *prn*, pertactin; *fim*, fimbrial antigen; *ptx*, pertussis toxin; ISE, IS element; NA, not applicable.

†New MLVA types identified in isolates by using the 8-loci MLVA scheme and corresponding MLVA types identified by using the 6-loci scheme are shown in parentheses.

‡No ISE or other disruptions were found in *prn*; IS*481*F/IS*481*R, IS*481* inserted in the forward/reverse direction; IS*1002*, IS*1002* disruption; HPT-G, homopolymeric tract variation.

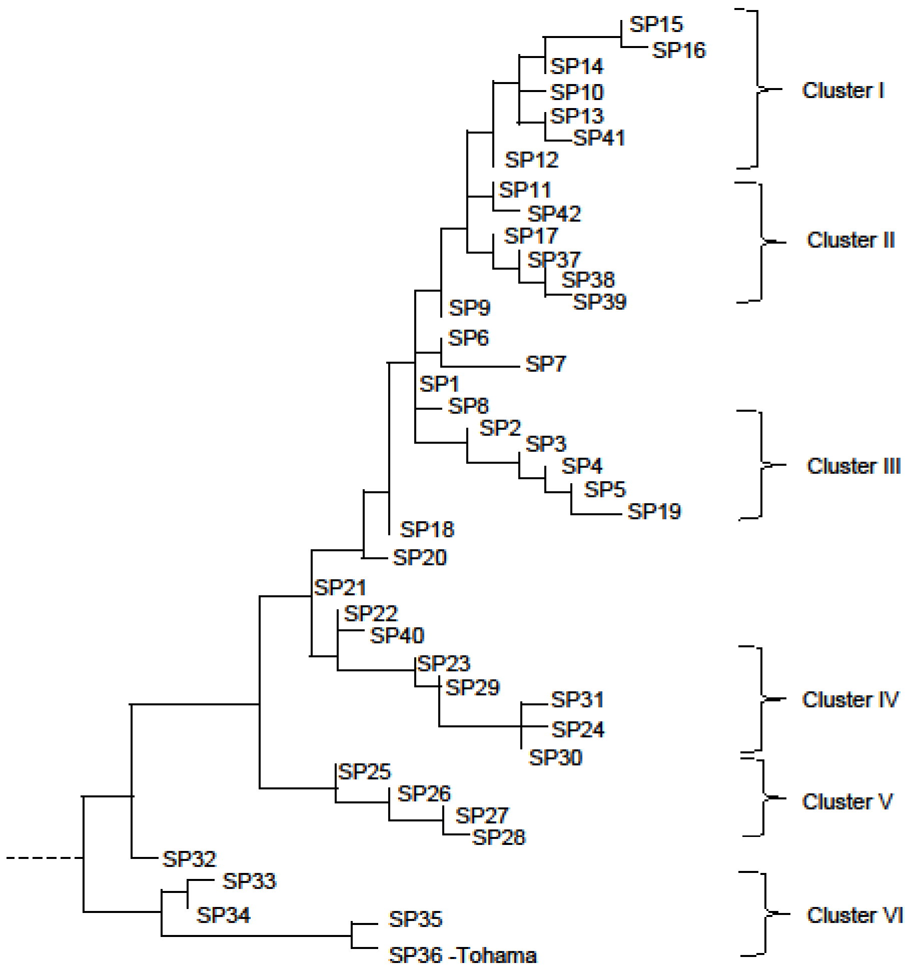

Technical Appendix Figure 1. Relationships of *Bordetella pertussis* isolates by using single-nucleotide polymorphism (SNP) typing, Australia, 2008–2012. Maximum-parsimony tree showing evolution of *B. pertussis* on the basis of 65 SNPs. Each branch is represented by a SNP profile (SP), which were grouped into clusters I–VI.

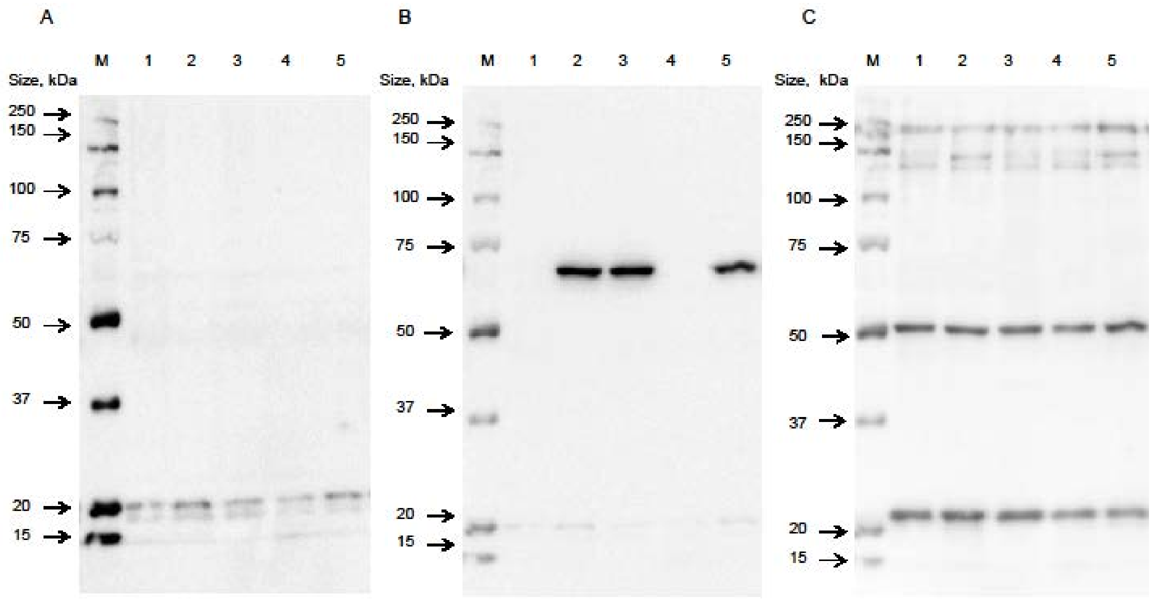

Technical Appendix Figure 2. Western blots of 5 *Bordetella pertussis* isolates treated with mouse polyclonal antibodies, Australia, 2008–2012. A) antibody against pertussis toxin (ptx), B) antibody against pertactin (prn), and C) antibody against filamentous hemagglutinin (fha). Lanes 1–5 in each panel correspond to isolates L1429, 1430, L1431, L1432, and Tohama I, respectively. Lanes M indicate molecular weight markers. All isolates showed positive results for ptx and fha. Only L1430 and L1431 expressed prn, and L1429 and L1432 did not express prn. Expected sizes are 21–23 kDa for ptx subunits, 69 kDa for prn, and 25 kDa, 50 kDa, and 125 kDa for fha.

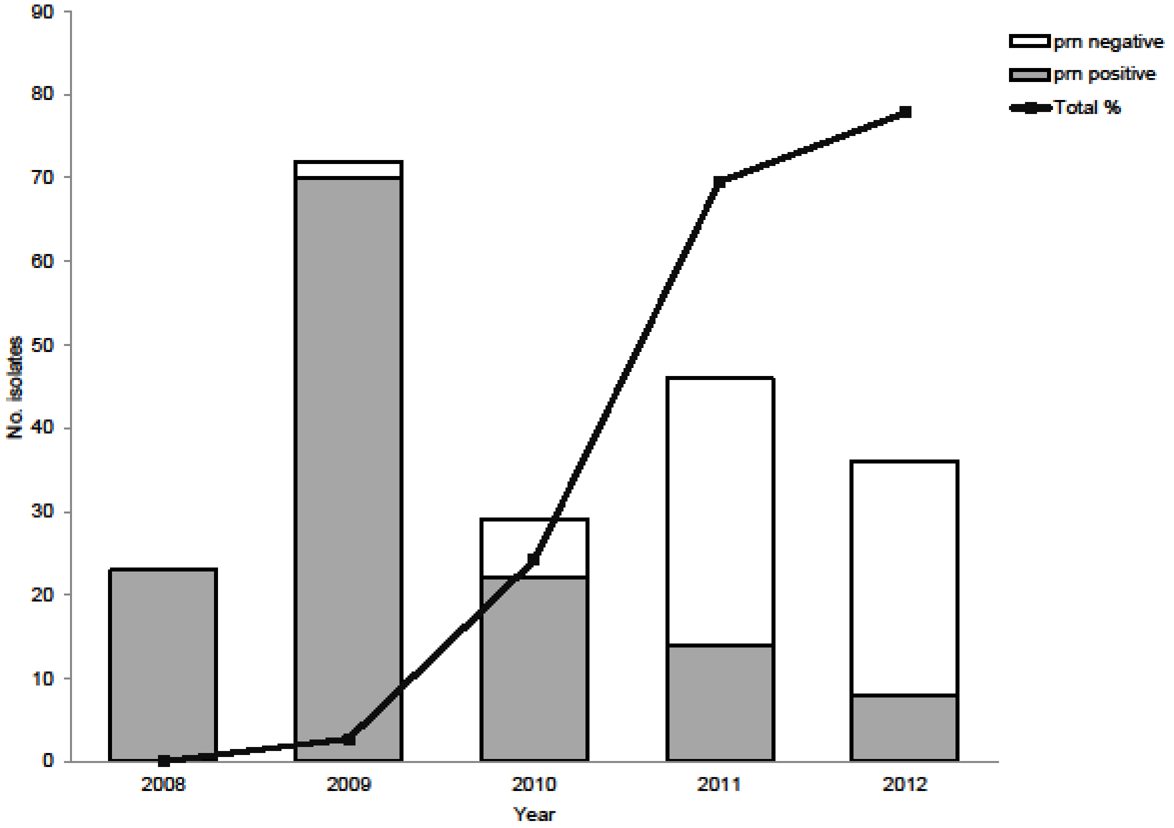

Technical Appendix Figure 3. Number and percentage of pertactin (prn)–negative *Bordetella pertussis* isolates in 2 states of Australia (New South Wales and Western Australia), 2008–2012.
